# Prognostic value of semi-quantitative parameters of ^18^F-FDG PET/CT in newly diagnosed multiple myeloma patients

**DOI:** 10.1007/s12149-022-01812-x

**Published:** 2022-12-17

**Authors:** Baoyu Wan, Song Zhang, Peng Wang, Pengyi Deng, Wenli Dai

**Affiliations:** 1 Department of Nuclear Medicine, Xinqiao Hospital, Third Military Medical University (Army Medical University), Chongqing, 400037 China; 2 Department of Nuclear Medicine, The First College of Clinical Medical Science, China Three Gorges University, Yichang, 443000 Hubei China

**Keywords:** Newly diagnosed multiple myeloma, ^18^F-FDG PET/CT, aMTV, aTLG, Prognosis

## Abstract

**Objective:**

To investigate the prognostic value of fluroine-18 fluorodexyglucose positron emission tomography/computed tomography (^18^F-FDG PET/CT) semi-quantitative parameter in newly diagnosed multiple myeloma (NDMM) and to design a new staging system including ^18^F-FDG PET/CT semi-quantitative parameters for NDMM.

**Methods:**

A total of 38 NDMM patients who underwent ^18^F-FDG PET/CT examination in Yichang Central People’s Hospital from February 2014 to April 2021 were collected. The relationship between the characteristics of ^18^F-FDG PET/CT (metabolic tumor volume of all lesions (aMTV), total lesion glycolysis of all lesions (aTLG), maximum standardized uptake values (SUVmax) of the lesion with largest MTV (mSUVmax), extramedullary disease (EMD), focal lesions (FLs)), the laboratory parameters, and prognostic parameters (progression-free survival (PFS) and overall survival (OS)) were analyzed retrospectively. SPSS 25.0 statistical software was used for statistical processing, Kaplan–Meier method was used for survival analysis, Log-rank method was used for univariate analysis, and Cox proportional risk model was used for multivariate analysis.

**Results:**

Univariate analysis showed that aMTV ≥ 90.97cm^3^, aTLG ≥ 283.31 g, hemoglobin (Hb) < 100 g/L, focal lesions (FLs) ≥ 10, (percentage of circulating plasma cells (CPC%) ≥ 30%, creatinine (Cr) ≥ 177umol/L, lactic dehydrogenase (LDH) ≥ 250 g/L might be the adverse prognostic factors of PFS in patients with NDMM, all *p* < 0.05; aMTV ≥ 90.97 cm^3^, aTLG ≥ 283.31 g, Hb < 100 g/L, FLs ≥ 10, mSUVmax ≥ 5.8, the presence of extramedullary disease (EMD) and PCPs ≥ 30% may be adverse prognostic factors for OS in patients with NDMM, all *p* < 0.05. Multivariate regression analysis showed that aMTV ≥ 90.97 cm^3^ was an independent risk factor for PFS in NDMM patients, *p* < 0.05; aMTV ≥ 90.97 cm^3^, mSUVmax ≥ 5.8, and the presence of EMD were independent risk factors for OS in the NDMM patients, all *p* < 0.05. According to the multivariate analysis results of OS, the New stage (NS) was performed. The 3-year OS rates of stage I, stage II, and stage III in NDMM patients were 100.0, 53.5, and 32.1%, respectively, *p* = 0.000.

**Conclusion:**

aMTV can predict PFS and OS of NDMM patients better than other parameters. NS which combined with aMTV can predict OS of NDMM patients better and can provide an accurate and simple method for risk stratification of NDMM patients.

## Introduction

Plasma cell tumors are plasma cell malignancies characterized by the abnormal proliferation of primary malignant plasma cells in the bone marrow and the production of monoclonal immunoglobulins [1]. According to the difference of serum abnormal immunoglobulin (M protein), it can be divided into 8 types: IgG type, IgA type, light chain type, IgD type, IgM type, IgE type, biclonal or polyclonal type, and non-secretory type. The main clinical manifestations are CRAB symptoms, namely hypercalcemia, renal insufficiency, anemia, and bone disease [2]. 80–90% of patients with multiple myeloma will develop into bone disease. Although the treatment of MM has been greatly developed, MM is still an incurable disease with very different survival outcomes. Therefore, a simple and accurate systematic scoring method is urgently needed to evaluate the prognosis of patients and to improve the clinical management process of patients. At present, a number of studies have evaluated the factors affecting the prognosis of MM. These studies have shown the following: fluroine-18 fluorodexyglucose positron emission tomography/computed tomography (^18^F-FDG PET/CT) characteristics (such as EMD, focal lesions (FLs), maximum standardized uptake value (SUVmax), and so on) and a lot of laboratory indicators (such as hemoglobin (Hb), albumin (Alb), creatinine (Cr), blood calcium (Ca^2+^), lactate dehydrogenase (LDH), β_2_ microglobulin (β_2_-M), C-reactive protein(CRP), percentage of circulating plasma cells (CPC%), and so on) can more accurately evaluate the prognosis of patients [3–11].

SUVmax is the most widely used in PET/CT parameters. Metabolic tumor volume (MTV) is a lesion metabolic volume parameter calculated on the basis of SUVmax, which is obtained by volume segmentation of lesions with high FDG uptake by setting a threshold. Total lesion glycolysis (TLG) is the product of MTV and mean standardized uptake value (SUVmean), metabolic tumor volume of all lesions (aMTV), and total lesion glycolysis of all lesions (aTLG) integrate the internal information of all lesions, quantify the total metabolic tumor burden, and can more comprehensively reflect the proliferation capacity, metabolic volume, and metabolic activity of systemic tumors. SUVmax of the lesion with the largest MTV (mSUVmax) can more accurately reflect the SUVmax value at the maximum tumor burden than SUVmax.

At present, there are few related studies using aMTV, aTLG, and mSUVmax to predict the prognosis of MM patients. In this study, 38 patients with newly diagnosed multiple myeloma (NDMM) from the Central People's Hospital of Yichang were retrospectively analyzed to study the relationship between ^18^F-FDG PET/CT imaging parameters, clinical laboratory parameters, and prognosis.

## Materials and methods

### Case collection

According to the Declaration of Helsinki, the study has been approved by the Medical Ethics Committee of Yichang Central People's Hospital (also known as the first Clinical Medical College of Three Gorges University) with approval number 2021–063-01. NDMM patients admitted to Yichang Central People’s Hospital were collected from February 2014 to April 2021. A total of 78 patients who were diagnosed with MM according to the International Myeloma Working Group guidelines were included at first [2], patients who had a history of tumors, a history of surgery or trauma in the past year, or with diabetes, or were unable to collect relevant laboratory parameters were excluded, at last, the remaining 38 cases were included in the study. Among them, 25 were males and 13 were females, aged 42–80 years old, with an average age of 60.29 years and a median age of 60.5 years. As of April 2021, the median follow-up time is 25 months. Collect the patients' first symptoms and related clinical parameters before treatment, such as Hb, Alb, Cr, Ca^2+^, LDH, β_2_-M, CPC%, International Staging System (ISS), and Durie-Salmon (DS) Staging System.

### Method

#### ^18^F-FDG PET/CT imaging method

^18^F-FDG is produced by Japan’s Sumitomo Cyclotron HM-10HC, and ensuring each batch of drugs meets the requirements through strict quality control. The imaging equipment is Siemens Biograph mCT-64 PET/CT. Before the examination, the patient fasted for more than 6 h, was monitored the fasting blood glucose level, injected ^18^F-FDG intravenously in a quiet state, with the dose range of 3.7–5.55 MBq (0.10–0.15 mCi)/kg, and was instructed to drink more water and urinate. After the imaging agent is injected, the patient rests quietly for 40–60 min. Before the examination, the bladder was emptied, and then 300–500 mL water was drunk to fill the stomach. First, the patients underwent  CT full-body scan (voltage 120 kV, current through 3D automatic real-time control by milliampere technology, CT scan with a thickness of 5 mm), and then whole-body PET collection, with the scan range from the top of the head to the upper femur. The PET scan is generally 6–7 beds, each bed is collected for 1.5 min, and the entire scanning process is about 15 min. Attenuation correction is performed on the collected data. The PET/CT whole-body imaging adopts the ordered subset maximum expected value method (OS-EM) for image reconstruction and transmits it to the Syngo MI workstation for image fusion.

#### ^18^F-FDG PET/CT image analysis

**Fig. 1:**
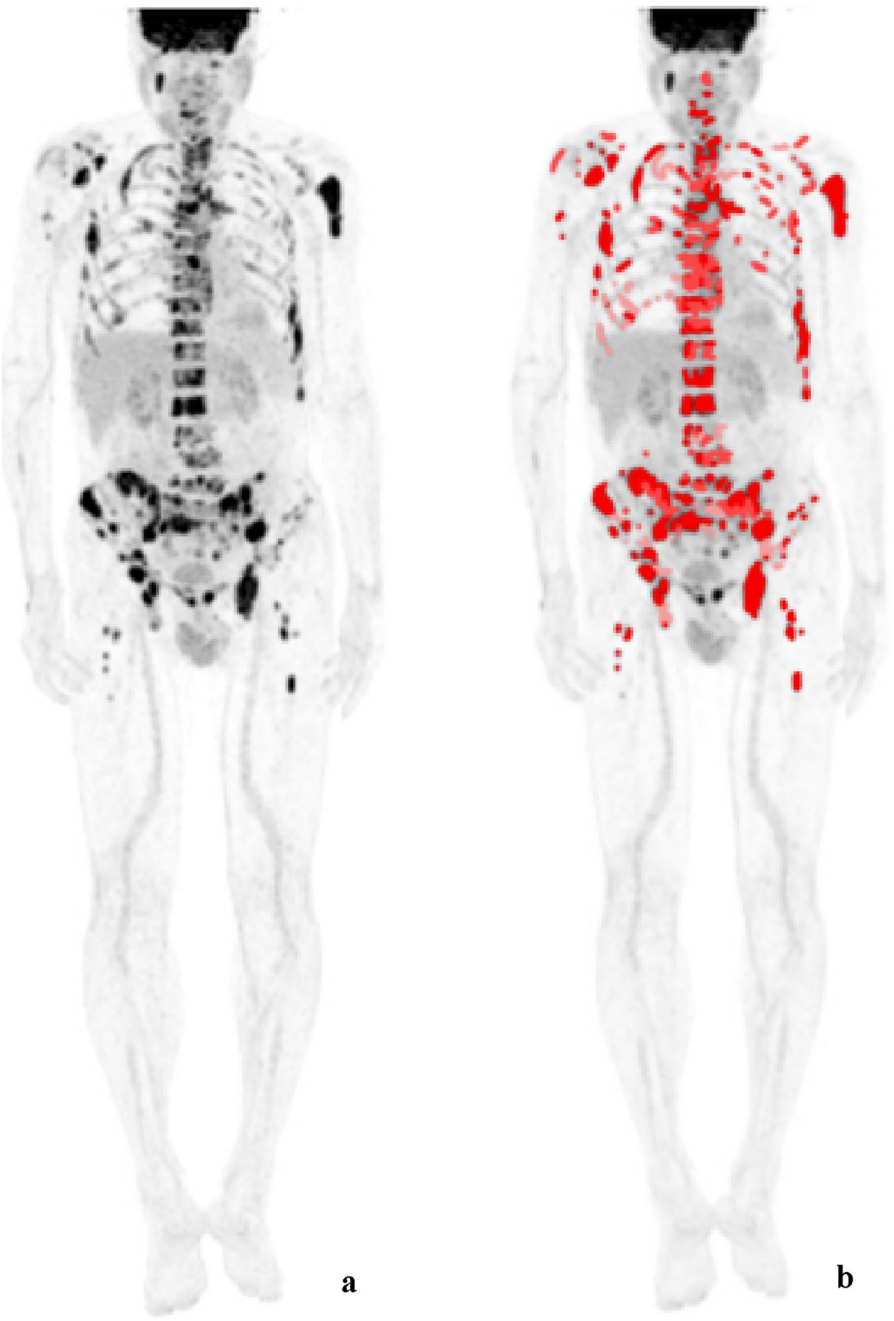
^18^F-FDG-PET/CT scan showing visual and volumetric parameters obtained in a MM patient. **a** Maximal intensity projection view. **b** All bone lesions in this patient are shown in red

The images were independently diagnosed by three experienced nuclear medicine physicians. When the diagnosis is inconsistent, the opinion of the majority shall prevail. The diagnostic criteria for a positive lesion are as follows: at least two consecutive layers of the lesion have a higher concentration of ^18^F-FDG imaging agent than the surrounding normal bone marrow tissue, or bone destruction on CT [12]. Bone benign lesions were excluded. The main observation indicators of PET/CT include the following: aMTV, aTLG, mSUVmax, the number of FLs, the location of bone lesions, whether there are pathological fractures (PF), and whether there is EMD. Taking each lesion SUV = 2.5 as the threshold, the computer software automatically outlines the region of interest (ROI) of the lesion and obtains the corresponding metabolic parameters: SUVmean and MTV. The aMTV of each patient is defined as the sum of the MTV of all individual lesions, and the TLG of each lesion is defined as the product of the MTV of the lesion and its corresponding SUVmean. The aTLG of each patient is defined as the sum of the TLG of all lesions. mSUVmax is defined as the maximum standard uptake value in the biggest MTV lesion. Examples of visual and volumetric parameters by ^18^F-FDG-PET/CT obtained in the same patient are reported in Fig. 1.

#### Treatment plan

The patients included in the study underwent standard treatment after ^18^F-FDG PET/CT examination and related laboratory examinations. Standardized treatment refers to the treatment of patients according to MM treatment norms or guidelines, including chemotherapy (such as thalidomide, lenalidomide, or bortezomib), autologous hematopoietic stem cell transplantation, or combination therapy.

#### Follow-up

Follow-up patients were with electronic records, telephone calls, and follow-up visits. Follow-up was until April 2021, with a median follow-up time of 25 months. The content of the follow-up included progression-free survival (PFS) and overall survival (OS) after standard treatment. PFS time is defined as the time from diagnosis to the first occurrence of disease progression, recurrence, death from any cause, or last follow-up. OS is defined as the time from diagnosis to death from any cause or the last follow-up.

### Statistical analysis

SPSS 25.0 was used for statistical processing. The ROC curve was used to calculate the maximum cut-off value of aMTV, aTLG, and mSUVmax to predict OS. Measurement data were expressed as mean ± standard deviation. Count data were expressed as rate, using *x*^2^ test. Comparison between groups was using Log-rank test. Univariate analysis was using Kaplan–Meier method. Cox proportional hazards model was used for multivariate analysis. The test level *α* = 0.05.

## Results

### Basic features

Among the 38 patients, 14 had back pain, 9 had dizziness, and 9 had chest pain. 15 were light chain types, 11 were IgG, 7 were IgA, and 3 were non-secretory types. According to DS staging, 5 cases are stage II and 33 cases are stage III; according to ISS staging, 6 cases are stage I, 15 cases are stage II, and 17 are stage III; 20 patients have anemia (Hb < 100 g/L), 21 patients had hypoproteinemia (Alb < 35 g/L), 7 patients had hypercalcemia (Ca^2+^  ≥ 2.65 mmol/L), 18 patients had high LDH (LDH ≥ 250 g/L), 9 patients had abnormal renal function (Cr ≥ 177 umol/L), 20 patients had high CRP (CRP ≥ 8.2 mg/L), 31 patients had β_2_M ≥ 3.5 mg/L, 15 patients had β_2_M ≥ 5.5 mg/L, the CPC% of 15 patients were ≥ 30%, and the basic characteristics of the patients are shown in Table 1.

**Table 1:** Basic characteristics of patients with NDMM

Characteristics	Mean ± SE/*n* (%)
Initial symptoms	
Dizziness and weakness	9(23.7)
Chest pain	9(23.7)
Lower back pain	14(36.8)
Others	6(15.8)
Age	60.3 ± 8.8
< 60	17(44.7)
≥ 60	21(55.3)
Sex	
Male	23(60.5)
Female	15(39.5)
DS stage	
I-I-I-II	5(13.2)
III	33(86.8)
ISS stage	
I-I-I-II	21(55.3)
III	17(44.7)
Hb	100.1 ± 29.5
< 100	20(52.6)
≥ 100	18(47.4)
Ca	2.2 ± 0.3
< 2.65	31(81.6)
≥ 2.65	7(18.4)
LDH	265.1 ± 148.1
< 250	20(57.9)
≥ 250	18(42.1)
CRP	17.0 ± 31.0
< 8.2	18(47.4)
≥ 8.2	20(52.6)
Cr	221.1 ± 422.2
< 177	29(76.3)
≥ 177	9(23.7)
Alb	35.1 ± 5.5
< 35	21(55.3)
≥ 35	17(44.7)
β_2_M	5.9 ± 2.7
< 3.5	7(18.4)
≥ 3.5	31(81.6)
< 5.5	23(60.5)
≥ 5.5	15(39.5)
CPC%	26.0 ± 18.4
< 30%	23(60.5)
≥ 30%	15(39.5)
Serum M protein	
IgA	7(18.4)
IgD	2(5.2)
IgG	11(28.9)
Light chain	15(39.5)
Non-secretory type	3(7.9)
FLs	
≥ 10	16(42.1)
< 10	22(57.9)
mSUVmax	7.3 ± 5.5
< 5.8	19(50.0)
≥ 5.8	19(50.0)
EMD	
Present	6(15.8)
Absent	32(84.2)
PF	
Present	11(28.9)
Absent	27(71.1)
aMTV	193.8 ± 259.1
< 90.97	20(52.6)
≥ 90.97	18(47.4)
aTLG	691.6 ± 1011.8
< 283.31	20(52.6)
≥ 283.31	18(47.4)

*NDMM* newly diagnosed multiple myeloma, *DS* Durie-salmon staging system, *ISS* International staging system, *Hb* hemoglobin, *Ca* blood calcium, *LDH* lactate dehydrogenase, *CRP* C-reactive protein, *Cr* creatinine, *Alb* albumin, *β*_*2*_*M* β2 microglobulin, *CPC%* percentage of circulating plasma cells, *FLs* focal lesions, *mSUVmax* maximum standardized uptake values of the lesion with largest MTV, *EMD* extramedullary disease, *PF* Pathological fracture, *aMTV* tumor metabolic volume of all lesions, *aTLG* total lesion glycolysis of all lesions

### ^18^F-FDG PET/CT features

**Fig. 2:**
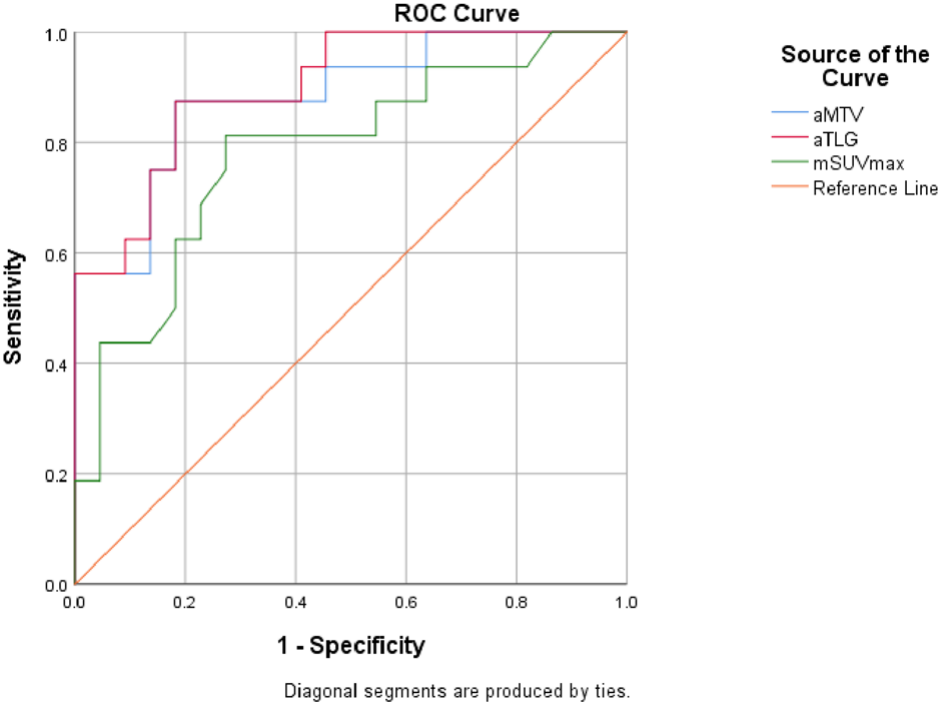
ROC curve results show that the best cut-off values of mSUVmax, aMTV, and aTLG are 5.8, 283.32 cm^3^, and 90.97 g, respectively

ROC curve results show that the best cut-off values of mSUVmax, aMTV, and aTLG are as follows: 5.8, 283.32 cm^3^, and 90.97 g, respectively, as shown in the Fig. 2. The range of bone lesion mSUVmax is 2.5–28.8, the median value is 5.8, and the average value is 7.3; the aMTV range is 0.8–1217.8 cm^3^, the median value is 79.1 cm^3^, and the average value is 193.8 cm^3^; the aTLG range is 2.3–4689.4 g, the median value is 242.7 g, and the average is 691.6 g. 16 of the 38 patients had FLs ≥ 10, 22 were < 10; 11 had pathological fractures, and 27 had not pathological fractures; 6 had EMD (3 cases were located in the parabone soft tissue, 1 in the soft tissue around the large joints of the limbs, 2 in the spinal canal). 32 cases had not EMD. The ^18^F-FDG PET/CT characteristics of the patients are shown in the Table 1.

### Univariate survival analysis to explore related factors affecting the prognosis of NDMM

Univariate analysis showed that aMTV ≥ 90.97 cm^3^, aTLG ≥ 283.31 g, Hb < 100 g/L, FLs ≥ 10, CPC% ≥ 30%, Cr ≥ 177 umol/L, LDH ≥ 250 g/L may be the adverse effects of PFS in this group of NDMM patients Prognostic factors, all *p* < 0.05; aMTV ≥ 90.97 cm^3^, aTLG ≥ 283.31 g, Hb < 100 g/L, FLs ≥ 10, mSUVmax ≥ 5.8, presence of EMD, CPC% ≥ 30% may be the adverse effects of OS in this group of NDMM patients, all *p* < 0.05, as shown in Table 2.

**Table 2:** Univariable analysis of factors predicting PFS and OS in patients with NDMM

Characteristics	*n*	PFS			OS		
		Mean	*χ*^*2*^	*p*	Mean * p*	*χ*^*2*^	*p*
Age							
< 60	17	31.078	0.098	0.754	40.761	0.541	0.462
≥ 60	21	32.311			36.898		
Sex							
Male	23	33.900	0.878	0.349	40.194	0.350	0.554
Female	15	28.113			37.700		
Hb							
< 100	20	23.844	7.174	0.007*	31.677	4.690	0.030*
≥ 100	18	41.430			46.035		
LDH							
< 250	20	39.616	5.406	0.020*	42.605	1.935	0.164
≥ 250	18	24.948			34.474		
CRP							
< 8.2	18	27.720	0.928	0.335	34.921	0.718	0.397
≥ 8.2	20	34.449			40.557		
Cr							
< 177	29	36.535	8.342	0.004*	42.257	3.450	0.063
≥ 177	9	18.111			29.361		
Ca							
< 2.65	31	30.791	0.501	0.478	38.600	0.003	0.958
≥ 2.65	7	37.500			40.375		
Alb							
< 35	21	37.158	1.662	0.197	42.580	1.473	0.225
≥ 35	17	28.228			36.106		
β2M							
< 3.5	7	34.714	0.288	0.591	35.714	0.115	0.734
≥ 3.5	31	30.664			39.026		
< 5.5	23	38.745	3.753	0.053	43.139	2.729	0.099
≥ 5.5	15	25.724			34.932		
CPC%							
< 30%	23	38.014	4.153	0.042*	45.665	5.862	0.015*
≥ 30%	15	25.507			32.101		
FLs							
≥ 10	16	47.446	10.521	0.001*	50.663	9.721	0.002*
< 10	22	24.151			31.625		
mSUVmax							
< 5.8	19	37.065	3.392	0.066	47.588	6.691	0.010*
≥ 5.8	19	26.430			28.848		
EMD							
Present	6	19.833	3.355	0.067	50.333	7.523	0.006*
Absent	32	31.581			30.382		
PF							
Present	11	27.003	1.044	0.307	42.205	2.358	0.125
Absent	27	33.912			29.980		
aMTV							
< 90.97	20	46.313	14.162	0.000*	52.056	15.363	0.000*
≥ 90.97	18	21.428			28.522		
aTLG							
< 283.31	20	41.880	7.305	0.007*	49.753	11.637	0.001*
≥ 283.31	18	24.000			29.962		

*p < 0.05, the difference was statistically significant

### Multivariate regression analysis was conducted to explore the relevant factors affecting the prognosis of MM

**Fig. 3:**
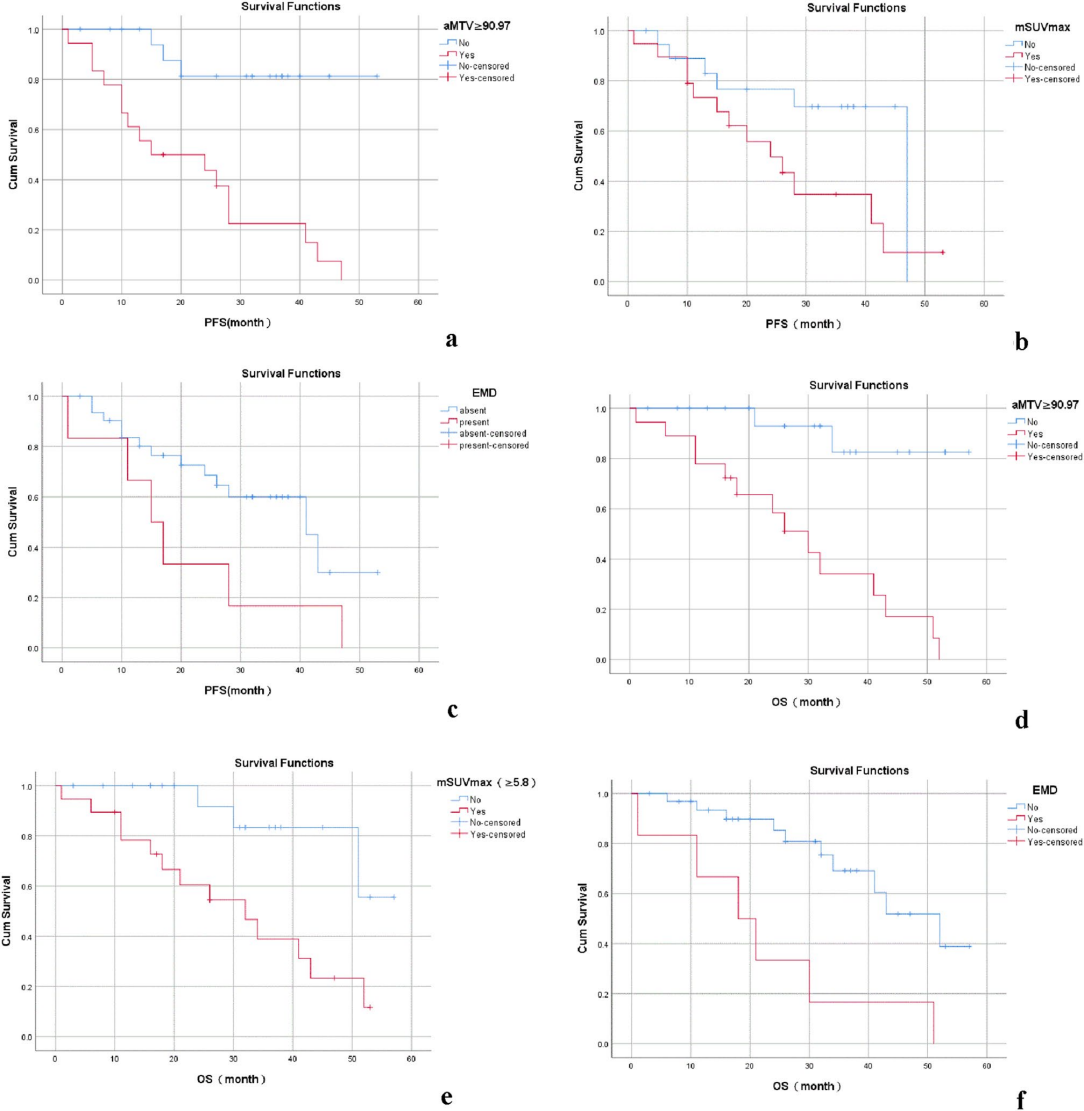
The results of Kaplan–Meier survival analysis for PFS and OS. **a** aMTV ≥ 90.97 cm^3^ was an independent risk factor for PFS, *p* < 0.05. **b**-**c** EMD and mSUVmax were not independent risk factors of PFS in NDMM patients, *p* > 0.05. **d**-**f** aMTV ≥ 90.97 cm.^3^, mSUVmax ≥ 5.8, EMD were independent risk factors for OS in this group of NDMM patients, *p* < 0.05

**Fig. 4:**
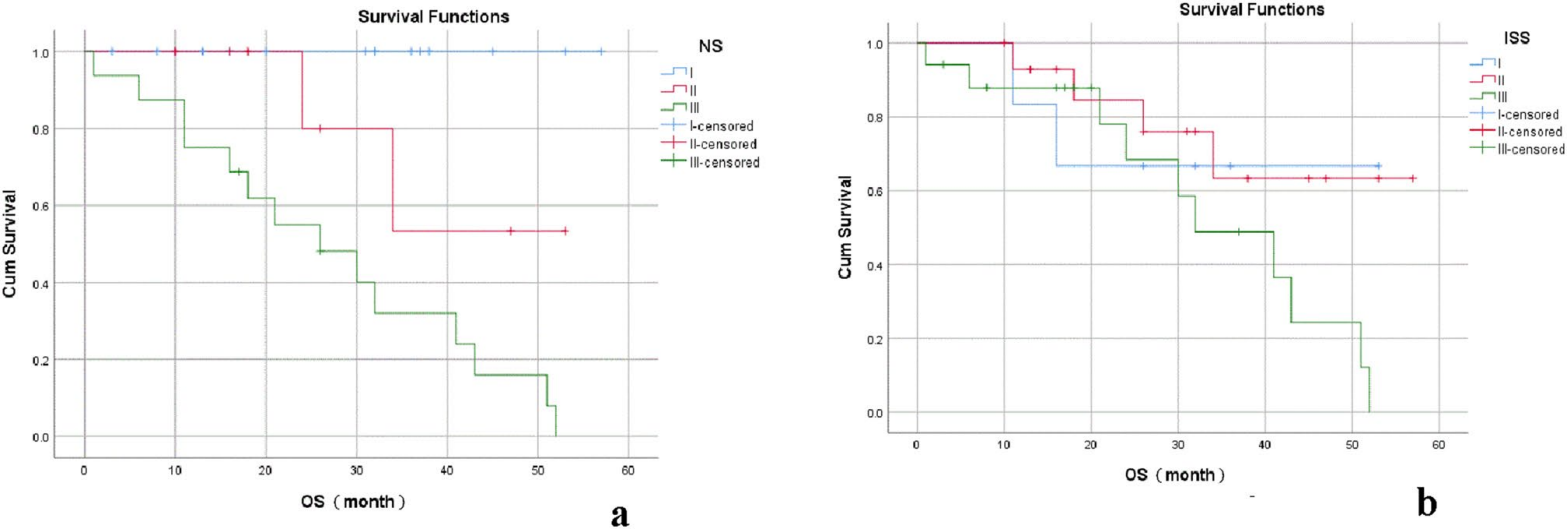
The results of Kaplan–Meier survival analysis for NS and ISS. The 3-year OS rates of patients with stage I, II, and III of the NS staging system were 100.0, 53.5, 32.1%, *p* = 0.000; the 3-year OS rates of patients with stage I, II, and III of the ISS staging system were 66.7, 63.3, and 48.8%, respectively, *p* = 0.145

Cox regression analysis was performed according to the results of univariate regression analysis. The results of Cox regression analysis showed that aMTV ≥ 90.97 cm^3^ was an independent risk factor for PFS in this group of NDMM patients (HR: 18.124, 95%CI: 1.179–278.632, *p* = 0.038); aMTV ≥ 90.97 cm^3^ (HR: 82.554, 95%CI: 2.333–2921.379, *p* = 0.038), mSUVmax ≥ 5.8 (HR: 23.095, 95%CI: 2.808–198.916, *p* = 0.003), EMD (HR: 7.918, 95%CI: 1.578–39.733, *p* = 0.012) are independent risk factors for OS in this group of NDMM patients, as shown in Table 3 and Fig. 3. According to the results of multivariate analysis of OS, a new stage (NS) was performed. Those with “aMTV ≥ 90.97 cm^3^, mSUVmax ≥ 5.8, and EMD” were all negative as stage I, 1 positive as stage II, and 2 or 3 positives are recorded as stage III. The 3-year OS rates of patients with stage I, II, and III of the NS staging system were 100.0, 53.5, and 32.1%, *p* = 0.000; the 3-year OS rates of patients with stage I, II, and III of the ISS staging system were 66.7%, respectively, 63.3, 48.8%, *p* = 0.145, as shown in Table 4 and Fig. 4.

**Table 3:** Multivariable analysis of factors predicting PFS and OS in patients with NDMM

Characteristics	PFS				Characteristics	OS		
	HR		95%CI	*p*		HR	95%CI	*p*
Hb					Hb			
< 100	2.354		0.688–8.048	0.172	< 100	3.769	0.836–16.987	0.084
≥ 100					≥ 100			
CPC%					CPC%			
< 30%	1.332		0.466–3.808	0.593	< 30%	2.248	0.618–8.182	0.219
≥ 30%					≥ 30%			
FLs					FLs			
≥ 10	1.919		0.262–14.034	0.521	≥ 10	.841	0.113–6.263	0.886
< 10					≥ 10			
aMTV					aMTV			
< 90.97	18.124		1.179–278.632	0.038*	< 90.97	82.554	2.333–2921.379	0.015*
≥ 90.97					≥ 90.97			
aTLG					aTLG			
< 283.31	0.161		0.019–1.362	0.094	< 283.31	0.086	0.004–1.661	0.104
≥ 283.31					≥ 283.31			
LDH					mSUVmax			
< 250	0.947		0.290–3.094	0.928	< 5.8	23.095	2.808–198.916	0.003*
≥ 250					≥ 5.8			
Cr					EMD			
< 177	1.645		0.512–5.287	0.403	Present	7.918	1.578–39.733	0.012*
≥ 177					Absent

**Table 4:** Comparison of OS between NS and ISS in patients with NDMM

Stage	NS				ISS			
	*n*	3 year OS(%)	*χ*^*2*^	*p*	*n*	3 year OS(%)	*χ*^*2*^	*p*
I	14	100.0			6	66.7		
II	8	53.5	17.956	0.000*	15	63.3	3.868	0.145
III	16	32.1			17	48.8		

*NS* new staging, *ISS* International staging system

## Discussion

At present, the commonly used inspection methods mainly include X-ray, CT, MRI, PET/CT and so on. The European Myeloma Network and European Society for Oncology guidelines recommend whole-body low-dose CT as the imaging method of choice for the initial evaluation of MM-related osteolytic lesions. MRI is the gold standard imaging method for detecting bone marrow involvement. PET/CT can provide valuable prognostic data and is the preferred technique for predicting and evaluating treatment response so far [12]. X-ray, CT, and MRI all have certain limitations. They can only judge the extent or size of the lesion but cannot judge the metabolism of the lesion. ^18^F-FDG PET/CT, as a safe and non-invasive imaging examination method, can reflect changes in glucose metabolism before bone destruction occurs and can directly show the activity of the lesion. In addition, PET/CT is a whole-body inspection method and it can also greatly improve the detection rate of extramedullary lesions. For MM bone lesions, PET/CT can identify more than 90% of osteolytic lesions and 6% of osteogenic lesions [13], with high sensitivity and specificity. PET/CT semi-quantitative parameters (aMTV and aTLG) have the advantages of reflecting biological information (such as the degree of metabolism and proliferation of tumor cells) and the volume of systemic tumors. They can comprehensively reflect the location and activity of systemic tumor lesions by evaluating the level of glucose metabolism. mSUVmax is the SUVmax value of the largest tumor burden lesions. They can better evaluate the tumor burden of NDMM patients than conventional PET parameters. There have been many studies applying PET/CT to the prognostic evaluation of MM. Their research has shown that patients with FLs ≥ 3, EMD, and high FDG intake have lower PFS and OS [4, 5, 7, 9–11, 14–16]. A total of 192 patients were included in the study by Ntambi et al. The results showed that the prognosis of MM patients with TLG > 620 g and MTV > 210 cm^3^ was worse, all *p* < 0.05 [35]. The study of Fonti et al. showed that patients with MTV ≥ 42.2 mL had shorter PFS than MTV < 42.2 mL (*x*^2^ = 3.9, *p* = 0.04), and patients with MTV ≥ 77.6 mL had shorter OS than MTV < 77.6 mL (*x*^2^ = 56.3, *p* < 0.0001) [36]. Fonti et al. obtained more accurate results after a longer follow-up of MM patients: MM patients with MTV ≤ 39.4 mL had better PFS and OS than patients with MTV > 39.4 mL (*p* = 0.0004, *p* = 0.0001). The results of our study showed that PFS of NDMM patients with aMTV ≥ 90.97 cm^3^ was 18.124 times that of patients with aMTV < 90.97cm^3^, *p* = 0.038; OS of NDMM patients with aMTV ≥ 90.97 cm^3^ was 82.554 times that of patients with aMTV < 90.97 cm^3^, *p* = 0.038; The OS of NDMM patients with mSUVmax ≥ 5.8 was 23.095 times that of patients with mSUVmax < 5.8, *p* = 0.008; the OS of NDMM patients with EMD was 7.918 times that of patients without EMD, *p* = 0.012. In fact, aMTV shows tumor metabolic burden of whole body, and probably because of this intrinsic feature, it surpasses the prognostic value of some visual parameters of 18F-FDG PET/CT, such as SUVmax and mSUVmax. In this study, “patients with aTLG ≥ 283.31 g have a worse prognosis” is only statistically significant in univariate analysis, while in multivariate analysis, there is no significant correlation between aTLG and prognosis, which may be due to the small sample size. Further verification will be carried out by increasing the sample size in future.

There are currently 4 staging systems commonly used to assess the prognosis of MM, including ISS, DS, Revised International Staging System, and Durie-Salmon Plus. ISS divides patients into three different groups based on β_2_M and serum albumin levels. DS combines the number of X-ray osteolytic lesions with a variety of clinical factors (such as serum calcium, hemoglobin levels, the number of M proteins, and renal function) for staging [17]. Revised International Staging System is based on the ISS combined with LDH and cytogenetics (t(14;16), t(4;14), del17p) for abnormal staging, which improves the prognostic value of the ISS staging system [18]. Durie-Salmon Plus is based on the number of lesions in ^18^F-FDG PET/CT for staging and is divided into subtypes A and B based on whether EMD and Cr > 2.0 mg/dL [17]. The study by Deng et al. showed that compared with DS, DS Plus and Revised International Staging System have better potential in the characteristics and stratification of MM patients [19]. In the study of Hu et al., they found that β_2_M, LDH, FLs, and SUVmax are independent factors that predict OS in MM patients, and DS staging cannot predict OS in MM patients. They proposed a new staging system based on β_2_M, FLs, SUVmax, and LDH. ISS and new staging system have shown great ability to distinguish between patients with poor prognosis and patients with good prognosis. However, ISS cannot distinguish the prognosis of stage II and stage III patients (*p* = 0.226). The results show that compared with ISS, NSS is a better prognostic model for OS in MM patients [11]. The study by Abe et al. combined ^18^F-FDG PET/CT with the proportion of CPC as a risk model for predicting the risk of newly diagnosed MM patients and divided the patients into three groups: PET-CPC stage I is no high-risk PET/CT Patients with low expression of CPC, stage III are patients with high-risk PET/CT performance and high CPC levels, and the remaining patients are classified as stage II. The conclusion of their study is that the three stages patients have significant differences in PFS and OS (*p* = 0.001). The PET-CPC staging system can predict the survival outcome of newly diagnosed MM patients [20]. Although the new staging system of Abe et al. and Hu et al. can predict the prognosis of patients well, they did not include aMTV and mSUVmax in the new staging system. This study included aMTV, mSUVmax, and EMD to develop a new staging system. The prognosis system can better evaluate the prognosis of patients than the ISS staging system: the 3-year OS rate in the NS staging system is 100.0, 53.5, and 32.1%, *p* = 0.000; the 3-year OS rate in the ISS staging system is 66.7, 63.3, and 48.8%, respectively, *p* = 0.145.

In recent years, cytogenetics and other indicators are also of great significance to the prognosis of MM. FISH has become a routine test for the prognostic stratification of MM patients. The characteristic genetic indicators are commonly superdiploid, 17p deletion, 1q21 amplification, and translocation Wait. For example, t(4;14), t(14;16), and 17p deletions are often considered to be related to the poor prognosis of MM patients. Yuan Jian et al. retrospectively analyzed the genetic data of 229 newly treated MM patients, and the results showed that 17p deletion, t(4;14), and 1q21 amplification are poor prognostic factors for MM patients [21]. Since this study is a retrospective study and the economic conditions of patients are limited, only a small number of patients underwent FISH test. This study still needs to further expand the sample, incorporate genetic indicators into the prognostic analysis, and further combine PET/CT imaging indicators and laboratory parameters to achieve the fusion of genetics, imaging, and laboratory parameters, which will help guide NDMM patients’ diagnosis, treatment, and prognosis.

### Conclusion

aMTV was better than other parameters, even SUVmax, in predicting PFS and OS in patients with NDMM. Compared with ISS, NS established in this study based on the results of Cox analysis can better evaluate the OS of MM patients. Although the new staging can more accurately predict the OS of MM patients to a certain extent, the limitation of this study lies in its retrospective nature and small sample, the selection of samples is biased, and the cytogenetic indicators have not been included, which is very important for verification. As a result, a large-sample prospective study still needs to be further confirmed.

## Data Availability

Data are available on request to the first author.

## Abbreviations

MM: Multiple myeloma
SBP: Solitary bone plasmacytoma
EMD: Extramedullary disease
^18^F-FDG PET/CT: Fluroine-18 fluorodexyglucose positron emission tomography/computed tomography
FLs: Focal lesions
SUVmax: Maximum standardized uptake values
Hb: Hemoglobin
Alb: Albumin
Cr: Creatinine
Ca: Blood calcium
LDH: Lactate dehydrogenase
β_2_M: β_2_ Microglobulin
CRP: C-reactive protein
CPC: Circulating plasma cell
MTV: Metabolic tumor volume
TLG: Total lesion glycolysis
SUVmean: Mean standardized uptake value
aMTV: Metabolic tumor volume of all lesions
aTLG: Total lesion glycolysis of all lesion
mSUVmax: Maximum standardized uptake values of the lesion with largest MTV
NDMM: Newly diagnosed multiple myeloma
ISS: International staging system
DS: Durie-salmon staging system
PF: Pathological fracture
ROI: Region of interest
PFS: Progression-free survival
OS: Overall survival
NS: New stage
